# Evaluation of Fear Using Nonintrusive Measurement of Multimodal Sensors

**DOI:** 10.3390/s150717507

**Published:** 2015-07-20

**Authors:** Jong-Suk Choi, Jae Won Bang, Hwan Heo, Kang Ryoung Park

**Affiliations:** Division of Electronics and Electrical Engineering, Dongguk University, 26 Pil-dong 3-ga, Jung-gu, Seoul 100-715, Korea; E-Mails: jjongssuk@dgu.edu (J.-S.C.); bangjw@dgu.edu (J.W.B.); gjghks@dgu.edu (H.H.)

**Keywords:** fear, nonintrusive multimodal measurement, facial temperature, subjective evaluation

## Abstract

Most previous research into emotion recognition used either a single modality or multiple modalities of physiological signal. However, the former method allows for limited enhancement of accuracy, and the latter has the disadvantages that its performance can be affected by head or body movements. Further, the latter causes inconvenience to the user due to the sensors attached to the body. Among various emotions, the accurate evaluation of fear is crucial in many applications, such as criminal psychology, intelligent surveillance systems and the objective evaluation of horror movies. Therefore, we propose a new method for evaluating fear based on nonintrusive measurements obtained using multiple sensors. Experimental results based on the *t-*test, the effect size and the sum of all of the correlation values with other modalities showed that facial temperature and subjective evaluation are more reliable than electroencephalogram (EEG) and eye blinking rate for the evaluation of fear.

## 1. Introduction

Emotion recognition has been researched in many fields, such as robotic systems and advanced driver assistance systems (ADASs) [1,2]. People can acquire user-specific services based on their emotional states, and the situation of being faced with unexpected danger can be predicted based on emotion recognition information. Thus, the accurate evaluation of emotion is becoming increasingly important in various fields. Previous research into the evaluation of emotion has largely been classified into two categories: single-modality-based methods and multimodal methods.

Single-modality methods measure emotion using visible-light cameras [3,4,5,6], thermal cameras [7], voice data [2,8,9] or physiological signals, such as from electrocardiography (ECG), electroencephalograms (EEGs) or skin temperature (SKT) data [10,11]. Camera-based methods have the advantage of providing greater convenience to users than other methods; in addition, the performance of the method is less affected by the movement of the head and body, because the sensors do not need to be attached to the body. However, visible-light camera-based methods cannot recognize emotions if the user makes an expressionless (neutral) face. In addition, visible-light camera-based methods have the disadvantage that the performance of facial feature point extraction can be affected by nonuniform illumination. It is difficult to detect the regions of facial features using thermal camera-based methods, because the textures of facial features are not distinct in the thermal image, which represents the difference of temperature on a face. Therefore, previously, researchers manually selected the facial feature regions when measuring the change of temperature according to emotion [7,12]. Other disadvantages of visible-light and thermal camera-based methods are their slower speed of data acquisition compared to physiological signal-based methods. The methods based on physiological signals generally use sensors attached to the skin. Agrafioti *et al.* studied a method of emotion detection based on ECG pattern analysis for various stimuli [10]. Although the physiological signal-based methods have the advantages of high accuracy of emotion detection and fast speed of data acquisition, user convenience is lower than that of camera-based methods, because of the attachment of sensors to the skin during measurement and because its performance is greatly affected by the movement of the head and body. All of these methods are based on a single modality; thus, their performance enhancement is limited.

Another approach, the multimodal-based method, has also been researched. Baumgartner *et al.* researched methods of measuring the change of emotion (elicited using pictures and classical music) based on various physiological signals [13]. Cheng *et al.* studied emotion recognition using photoplethysmography (PPG), electromyography (EMG), ECGs, galvanic skin response (GSR) and temperature [14]. Additionally, Chun *et al.* researched emotion recognition using various physiological signals, such as skin conductance (SC), blood volume pressure, skin temperature (ST), EMG and respiration [15]. These methods have the advantage of higher accuracy compared with single-modality-based methods, because the use of more than two modalities can increase the credibility of measurements. Most previous multimodal-based methods used various physiological signals; however, they have disadvantages in that the performance can be affected by head or body movements, and they can cause inconvenience to the user, because of the attachment of sensors to the body. Among various kinds of emotion evaluation, accurate evaluation of fear is crucial in many applications, such as criminal psychology, intelligent surveillance systems and the objective evaluation of horror movies.

In previous research [16], they proposed a system for real-time detection of fear. As the features for fear detection, they used the ratio of the slow to fast wave powers of the EEG signal when each user was watching the scary video. In [17], Schutter *et al.* used the ratio of the slow to fast wave powers of the EEG signal for predicting the emotional imbalances in reward- and punishment-driven behavior of diagnostic value for psychopathology. In the research of Putman *et al.* [18], they also showed the result that there exists a negative relation between the ratio of the slow to fast wave powers of the EEG signal and fear. In previous research [19], they measured the participant’s response to angry facial expressions by functional magnetic resonance imaging (fMRI) and analyzed that neural responses to angry facial expressions can be interpreted in terms of fear. All of this research used the single modality of EEG to measure fear, and the measurement by fMRI can be limited in various experimental environments due to its high cost and the size of the device. Therefore, we propose a new method for evaluating fear using nonintrusive multimodal measurements. Our research is novel in the following three ways compared to previous works.

- First, to enhance the accuracy of the evaluation of fear, we measure electroencephalogram (EEG) signals, eye blinking rate (BR), facial temperature (FT) and a subjective evaluation (SE) score before and after a user watches a horror movie.
- Second, to accurately and conveniently measure the blinking rate of each user, a remote eye image capturing system is implemented using a high-speed mega-pixel camera. In addition, the changes in facial temperature are non-intrusively measured using dual (visible-light and thermal) cameras; the region of interest (ROI) for measuring the changes of facial temperature on the face are automatically detected in successive images, which enables enhancing the measurement accuracy of evaluating fear.
- Third, we prove that our experimental results are caused by fear through the comparative experiments with a video clip having the same length and emotionally-neutral content presented to the subjects. In addition, we compensate for the measured values with the horror movie by using those with the video clip of emotionally-neutral content in order to obtain more accurate values in the experiments for measuring the fear emotion excluding other factors.

Table 1 indicates the comparison of previous research of emotion recognition to our method.

The remainder of this paper is organized as follows. In Section 2, we describe the proposed method and all of the data analysis methods. Section 3 presents the experimental environment and results. Analyses and discussions are shown in Section 4. Finally, we present our conclusions in Section 5.

**Table 1:** Comparison between the previous methods and the proposed method of emotion recognition.

Category		Method	Advantages	Disadvantage
Using a single modality	Visible-light camera-based methods [3,4,5,6].	User’s emotion is recognized based on facial expression in an image.	- Providing comfort to the user without the attachment of sensors. - Less expensive method than bio-signal or thermal camera-based methods.	- Analyzing emotion is difficult if the person has no expression. - Extraction of the facial feature points can be affected by non-uniform illumination.
	Thermal camera-based methods [7,12].	Measuring the change of facial temperature according to emotion.	- Providing comfort to the user without the attachment of sensors. - Analyzing emotion is easy, even if the person has no expression. - Extraction of the facial feature points is not affected by illumination condition.	- More expensive method than visible-light camera-based method. - Difficult to detect regions of facial features because the texture of facial features is not distinct in the thermal image.
	Voice-based methods [2,8,9].	Measuring the change of voice features according to emotion.	Less expensive method than bio-signal or thermal camera-based method.	- The performance can be affected by surrounding noises.
	Physiological signal-based methods [10,11,16,17,18].	ECG [10] and EEG [11,16,17,18] are analyzed for emotion detection.	High accuracy of emotion detection and fast speed of data acquisition.	- More discomfort to the user because sensors are attached to the body. - More influenced by the motion of the head, body and muscles than camera- or voice-based methods.
Using multiple modalities	Multiple physiological signal-based methods [13,14,15].	- Using EEG, heart rate, SC, respiration, ST and psychometrical ratings [13]. - Using PPG, EMG, ECG, GSR and ST [14]. - Using SC, blood volume pressure, ST, EMG and respiration [15].	Higher accuracy of emotion detection compared to single modality-based methods.	- More discomfort to the user because many sensors are attached to the body. - More influenced by the motion of the head, body and muscles than single modality-based methods.
	Hybrid method using both physiological signals and non-intrusive camera-based methods (proposed method).	Using facial temperature, EEG, blinking rate and subjective evaluation for evaluating fear.	- Higher accuracy of emotion evaluation compared to single modality-based methods. - More comfort to the user and higher freedom of movement without the attachment of sensors, except for a wireless EEG device. - Facial feature positions on the thermal image can be easily detected using dual (visible-light and thermal) cameras.	Larger amount of data to be processed after acquiring the image sequences by dual cameras.

## 2. Evaluating Fear by Multimodality-Based Measurements

### 2.1. Overall Procedure of the Proposed Method and Multimodal Sensors for Measuring Fear

**Figure 1:**
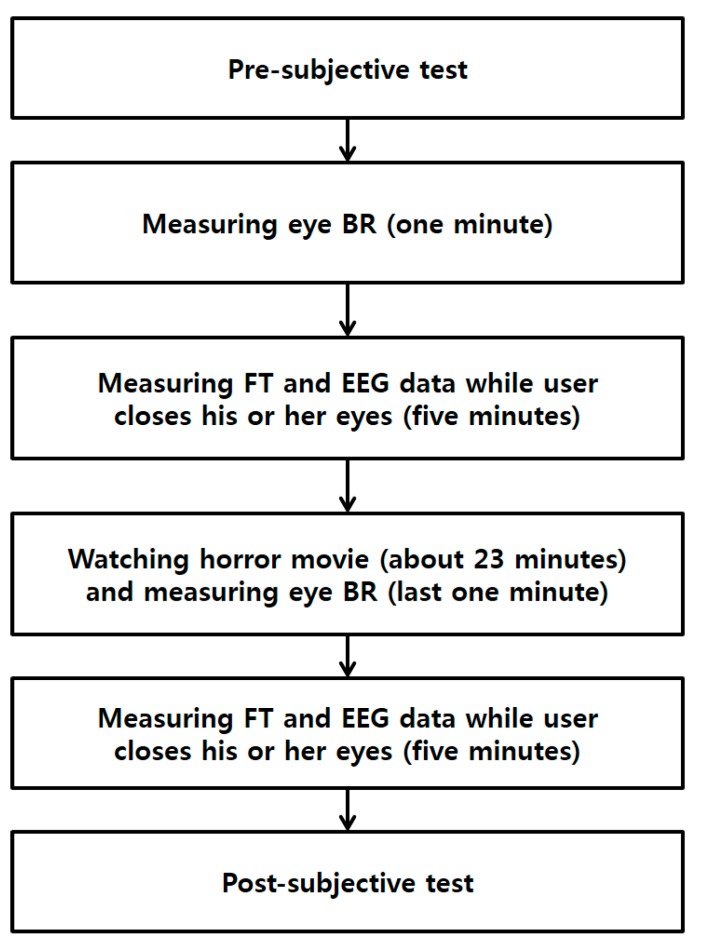
Flowchart of the experimental procedure of our research (BR is blinking rate and FT is facial temperature).

We conducted data acquisition experiments, as outlined in Figure 1. In our method, four types of data are acquired before a user watches a horror movie. First, we perform subjective evaluation with the user to determine his or her condition. Then, we measure the eye blinking rate of the user with a high-speed camera for 1 min. In addition, the user is requested to close his or her eyes to minimize external stimuli while measuring EEG and facial temperature for 5 min. Then, the user watches a horror movie for 23 min. To maximize the user’s fear, we performed the experiment while the light is turned off and included the sound effects of the movie. In the last 1 min of the movie, the eye blinking rate is measured again, for comparison with the measurement before watching the movie. After finishing the movie, we ask the user to close his or her eyes, and facial temperature and EEG data are measured again for 5 min. Finally, subjective evaluation is conducted again to check his or her condition after watching the movie.

**Figure 2:**
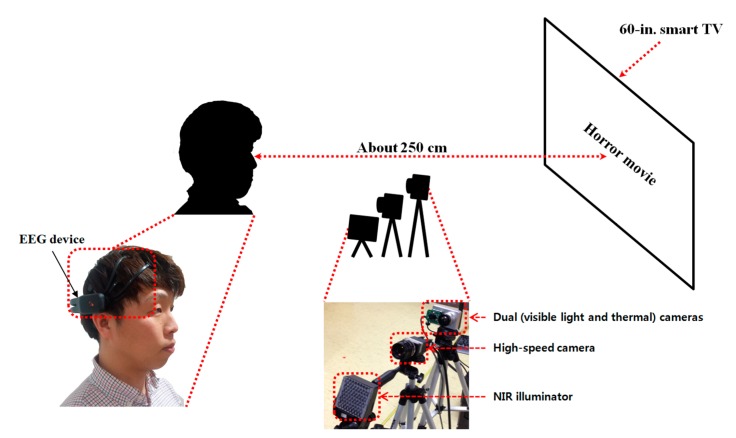
Proposed system for evaluating fear.

Figure 2 shows the experimental environment and system used in our research. The horror movie was displayed on a 60-in. smart TV with a resolution of 1920 × 1280 pixels. We obtained the EEG signals, facial temperature and eye blinking rate of subjects using an EEG device, dual (visible-light and thermal) cameras and a high-speed camera [20], respectively.

As shown in Figure 2, each subject watched the horror movie on a smart TV at a distance of approximately 250 cm. A near-infrared (NIR) illuminator was used with the high-speed camera, because the light is turned off while the user watches the movie in order to maximize the user’s fear. In addition, to measure the eye blinking rate, the accurate position of the pupil should be located, and this is typically distinct under NIR illumination. The NIR illuminator was composed of NIR LEDs of 850 nm [21], which has no dazzling effect on the user’s eye.

**Figure 3:**
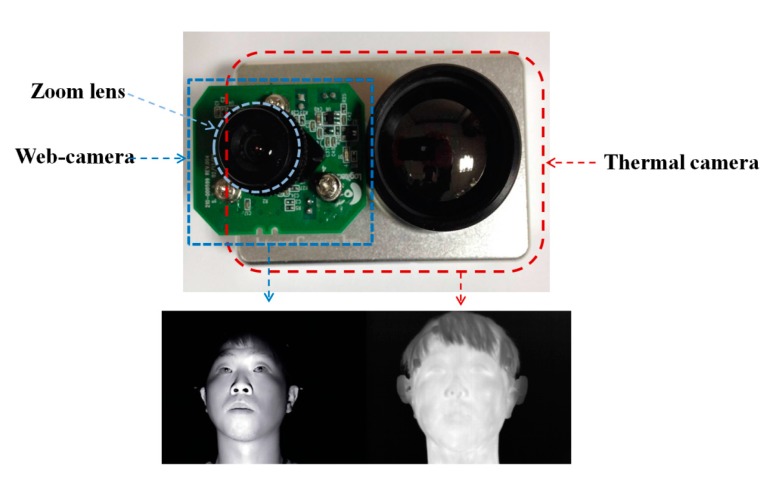
Dual (visible-light and thermal) cameras used in our method and their images.

As shown in Figure 3, we measured facial temperature using dual (visible-light and thermal) cameras. Previous research [22] reported that the heat energy from the human body and face can be observed in medium-wave IR (MWIR) and long-wave IR (LWIR) sub-bands. Therefore, these sub-bands are called thermal sub-bands, and we refer to images in these sub-bands as thermal images. We used a commercial thermal camera (ICI 7320 Pro) [23]. The spectral range of the thermal camera is from 7 to 14 μm, which corresponds to the upper range of MWIR and most of LWIR. The measurement accuracy of the temperature is ±1 °C. The image resolution is 320 × 240 pixels, and each pixel contains 14 bits of data. This camera can measure a temperature range from −20 °C to 100 °C [23]. The field of view (FOV) of the thermal camera is 18° and 14° in the horizontal and vertical directions, respectively.

It is usually difficult to detect the regions of facial features in a thermal image because the textures of facial features are not distinct, as shown in Figure 3. Therefore, previous researchers manually selected the facial feature regions for measuring the change of temperature according to emotion [7,12]. To overcome this problem, we use dual cameras, as shown in Figure 3. A commercial web-camera (C600) is used as the visible-light camera [24]. The image resolution of the visible-light camera is 800 × 600 pixels. To obtain the image of the magnified face region, a zoom lens of 1.6-times magnification is attached to the web-camera. This camera was used to detect the regions of the face, eyes and nostrils. Because there is some positional disparity between the visible-light and thermal cameras, a geometric transform is used to compensate for this disparity. Based on this transform and the detected regions of facial features, the ROI in which the facial temperature is measured is defined in the thermal image. Further details are presented in Section 2.2.

**Figure 4:**
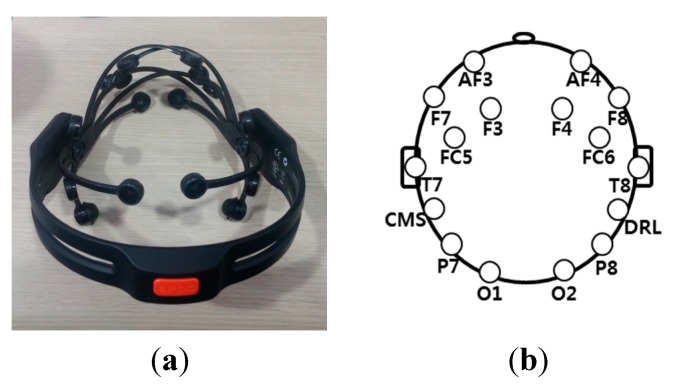
Commercial EEG device and locations of 16 electrodes based on the international 10–20 system. (**a**) Emotiv EPOC headset; (**b**) positions of 16 electrodes.

As shown in Figure 4a, we acquired EEG data using a commercial wireless-headset-type EEG device (Emotiv EPOC [25]). It is inexpensive, and it has 16 noninvasive electrodes. These 16 electrodes are composed of two reference electrodes (common mode sense (CMS) and driven right leg (DRL)) and 14 additional electrodes for acquiring EEG data. The location of the electrodes is based on the international 10–20 system of electrode placement, shown in Figure 4b [26]. The EEG signal is processed by the built-in digital fifth-order Sinc filter; the sampling rate is 128 Hz (128 samples/s) for each electrode. Through normalization, the DC levels of the measured EEG signals are adjusted; the range of the EEG signals is set from −1 to 1 using min-max scaling. The EEG signals are transformed into the frequency domain by a Fourier transform with a window length of 128 samples [27].

The high-speed camera was used to acquire eye images to determine eye blinking rate. Although this camera can capture images at a resolution of 2048 × 2048 pixels at 150 frames per second (fps) [20], the acquisition speed is actually much slower than 150 fps, because of the time taken by the computer to save the image. Therefore, we improved the image acquisition speed by saving a cropped version of the image of only 2048 × 512 pixels, which includes both eyes.

### 2.2. The Analysis of Facial Temperature Variation

**Figure 5:**
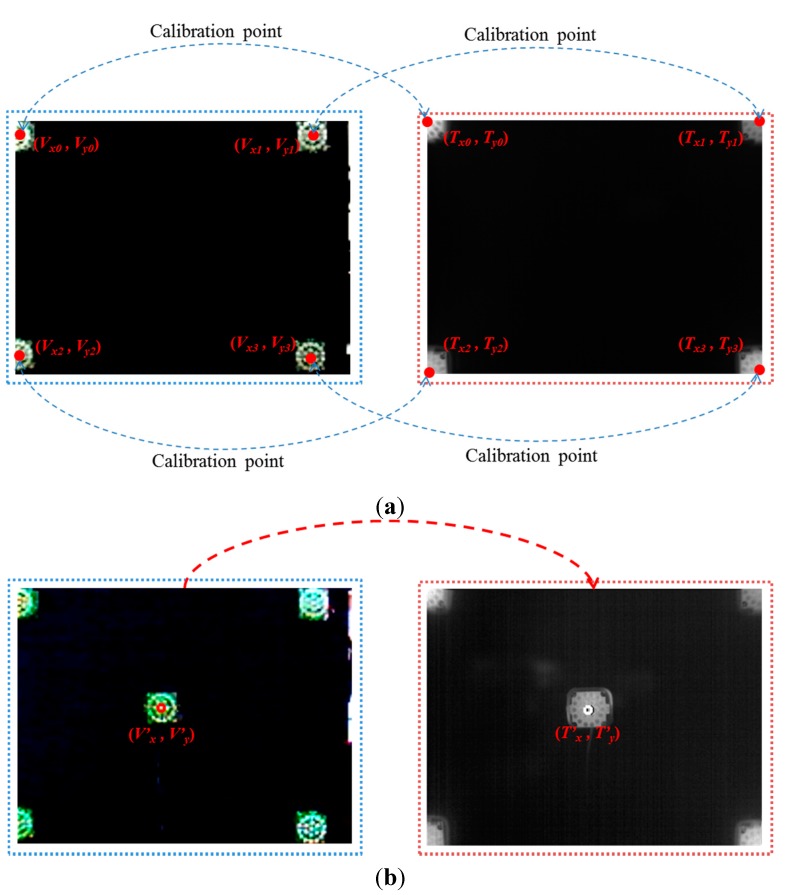
Four corresponding (calibration) pairs of points produced by four NIR illuminators to obtain the geometric transform matrix and an example for measuring calibration accuracy. (**a**) Four pairs of corresponding (calibration) points; (**b**) pair of points for measuring calibration accuracy.

As explained in Section 2.1 and shown in Figure 3, the images are acquired using dual cameras for measuring user facial temperature variations. It is usually difficult to detect the regions of facial features in the thermal image, because the textures of facial features are not distinct in the image, as shown in Figure 3. Therefore, facial feature regions are detected by the visible-light camera. However, the viewing angle and image resolution of the visible-light camera are different from those of the thermal camera. In addition, there exists a positional disparity between the visible-light and thermal cameras, as shown in Figure 3. Therefore, the coordinates of the detected facial feature regions in the visible-light image cannot be directly used in the thermal image. To solve this problem, the two axes of the visible-light and thermal cameras are parallel with the minimum horizontal distance between them, as shown in Figure 3. Then, we make the coordinates of the two images (visible-light and thermal) coincident using a geometric transform, as shown in Equation (1) and Figure 5.

(1) $$\left[\begin{array}{c} \begin{array}{cc} T_{\text{x}0} & T_{x1}\\ T_{y0} & T_{y1} \end{array}\;\;\;\;\begin{array}{cc} T_{x2} & T_{x3}\\ T_{y2} & T_{y3} \end{array}\\ \begin{array}{cc} 0\; & 0\\ 0\; & 0 \end{array}\;\;\;\;\;\;\;\;\begin{array}{cc} 0 & \;0\\ 0 & \;0 \end{array} \end{array}\right]=\left[\begin{array}{c} \begin{array}{cc} a & b\\ e & f \end{array}\;\;\;\;\begin{array}{cc} c & d\\ g & h \end{array}\\ \begin{array}{cc} 0 & 0\\ 0 & 0 \end{array}\;\;\;\;\begin{array}{cc} 0 & 0\\ 0 & 0 \end{array} \end{array}\right]\left[\begin{array}{c} \begin{array}{cc} V_{x0}\; & V_{x1}\\ V_{y0}\; & V_{y1} \end{array}\;\;\;\;\;\;\;\;\;\;\begin{array}{cc} V_{x2}\; & V_{x3}\\ V_{y2}\; & V_{y3} \end{array}\\ \begin{array}{cc} V_{x0}V_{y0} & V_{x1}V_{y1}\\ 1 & 1 \end{array}\;\;\;\;\;\begin{array}{cc} V_{x2}V_{y2} & V_{x3}V_{y3}\\ 1 & 1 \end{array} \end{array}\right]$$

(2) $$\left[\begin{array}{c} \begin{array}{c} T\prime_{x}\\ T\prime_{y} \end{array}\\ \begin{array}{c} 0\\ 0 \end{array} \end{array}\right]=\left[\begin{array}{c} \begin{array}{cc} a & b\\ e & f \end{array}\;\;\;\;\begin{array}{cc} c & d\\ g & h \end{array}\\ \begin{array}{cc} 0 & 0\\ 0 & 0 \end{array}\;\;\;\;\begin{array}{cc} 0 & 0\\ 0 & 0 \end{array} \end{array}\right]\left[\begin{array}{c} \begin{array}{c} V\prime_{x}\\ V\prime_{y} \end{array}\\ \begin{array}{c} V\prime_{x}V\prime_{y}\\ 1 \end{array} \end{array}\right]$$

In Equation (1), (*V*_x0_, *V*_y0_), … (*V*_x3_, *V*_y3_) are the four positions in the visible-light image, whereas (*T*_x0_, *T*_y0_), … (*T*_x3_, *T*_y3_) are the four corresponding positions in the thermal image. Using these four corresponding pairs of positions, the geometric transform matrix including the eight parameters of *a*, *b*, *c*, … *h* can be obtained using Equation (1). Then, the position of facial features (*T*’_x_, *T*’_y_) in the thermal image can be calculated using the geometric transform matrix with the detected position of facial features (*V*’_x_, *V*’_y_) in the visible-light image, as shown in Equation (2). To obtain the geometric transform matrix, four corresponding pairs of positions are necessary. Because an object that does not radiate heat does not appear in the thermal image, four NIR illuminators were used to indicate the four corresponding (calibration) pairs of points [(*V*_x0_, *V*_y0_), … (*V*_x3_, *V*_y3_)] and [(*T*_x0_, *T*_y0_), … (*T*_x3_, *T*_y3_)] in the visible light and thermal images, respectively, as shown in Figure 5.

**Figure 6:**
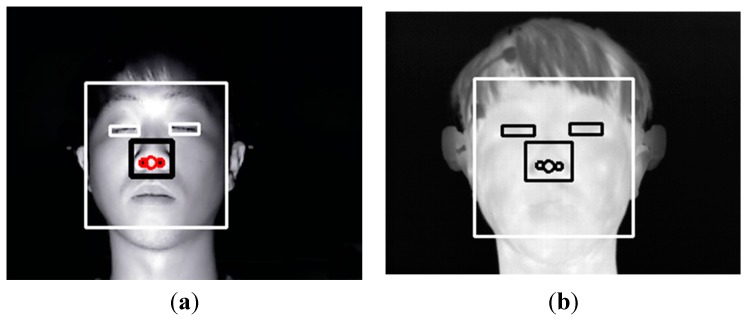
(**a**) Detected face and facial feature regions in the visible-light image; (**b**) mapped face and facial feature regions in the thermal image after geometric transformation.

Figure 6 shows the detected facial feature regions, such as the face, eyes, nose and nostrils in visible-light and thermal images. An adaptive boosting (AdaBoost) algorithm is used to detect the face region [28]. Then, the search areas of eyes and nose are defined within the detected face region. Within the search areas, the eyes and nose regions are located using the AdaBoost algorithm. Our method performs image binarization in the detected nose region to accurately detect both nostrils. Based on the detected nostrils, the center of the nose is detected. As explained in Section 2.1, the light is turned off while the user watches the movie to maximize the user’s fear. Therefore, additional NIR illuminators are used, as shown in Figure 2; the images from the visible-light camera are obtained under NIR illumination, and the NIR cutting filter inside the web-camera is replaced by an NIR passing filter.

The detected face and facial feature regions are indicated by the rectangular boxes and circles, respectively, in the visible-light image shown in Figure 6a. Figure 6b shows the mapped regions of the face and facial features in the thermal image (from the detected face and facial feature regions of Figure 6a) after using the geometric transform matrix of Equation (2).

**Figure 7:**
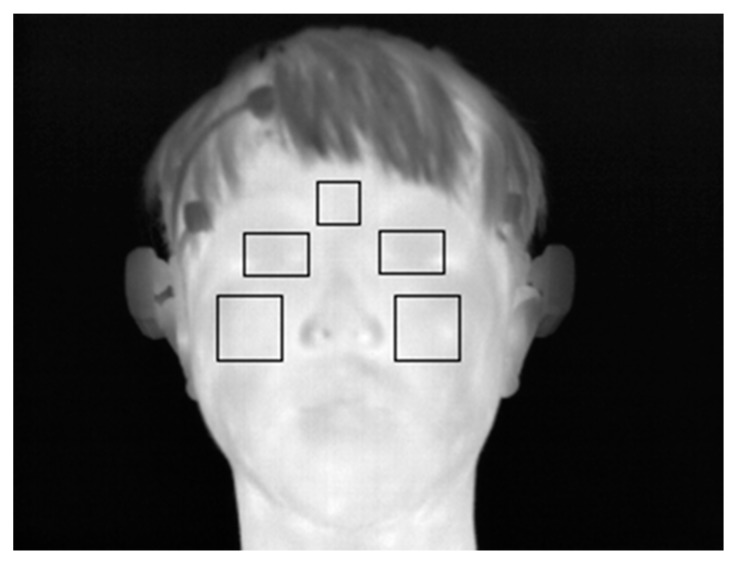
Example of defined ROIs used to measure the change of facial temperature.

Based on the detected facial feature regions, the ROIs for which the change of facial temperature is measured are defined. These ROIs are defined based on previous research [7,12]. In these studies, the ROIs for measuring the change of facial temperature in the face area in successive images were manually defined, which can cause the inconsistent definition of the ROIs and take a considerable amount of time [7,12]. Therefore, our method automatically detects the ROIs. Figure 7 shows the defined ROIs used to measure the change of facial temperature.

### 2.3. Analysis of EEG Variation

**Figure 8:**
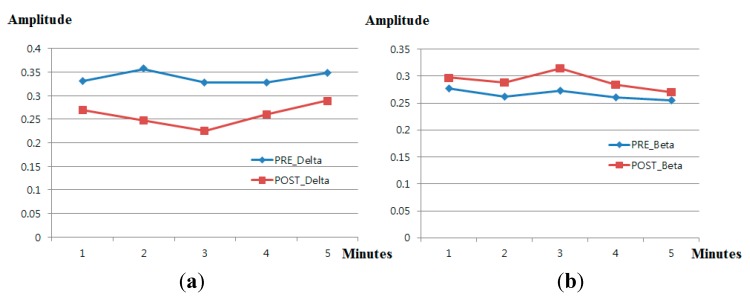

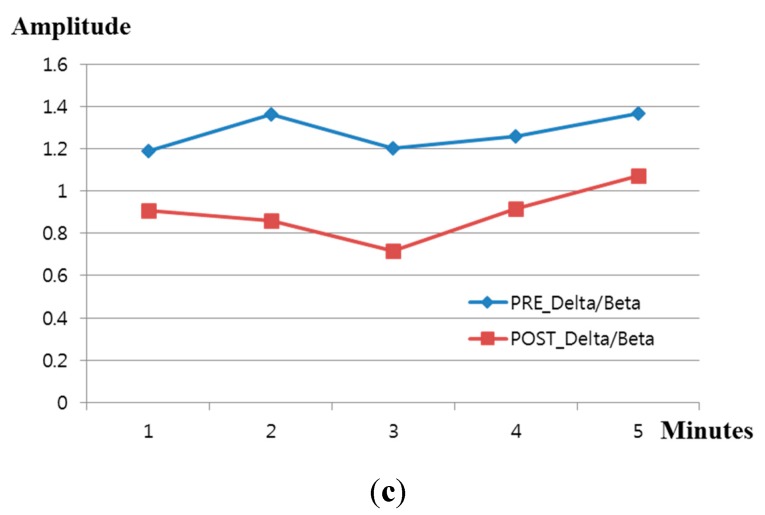
Comparison of delta and beta waves before and after watching a horror movie. (**a**) Change in delta waves; (**b**) change in beta waves; (**c**) change in the ratio of delta to beta waves.

In general, brain waves can be classified into several bands: delta waves (0.5–4 Hz), theta waves (4–8 Hz), alpha waves (8–13 Hz) and beta waves (>13 Hz) [16]. The delta wave is slower than alpha and beta waves, whereas the beta wave is faster than delta and alpha waves. Fear has previously been analyzed using the ratio of slow waves (delta waves) to fast waves (beta waves) from EEG data [16,17,18,19]. In addition, other various emotions were analyzed using the ratio of slow waves (delta waves) to fast waves (beta waves) [29,30,31]. Using this criterion, we analyzed the power ratio of delta band (0.5–4 Hz) to beta band (>13 Hz) from the EEG data to measure the fear emotion. Figure 8 shows the change of amplitude of delta and beta waves (at the O1 electrode) before (“PRE_Delta” and “PRE_Beta”) and after (“POST_Delta” and “POST_Beta”) watching the horror movie. As shown in Figure 8, because POST_Delta is less than PRE_Delta and POST_Beta greater than PRE_Beta, we can estimate that the power ratio of the delta band to the beta band decreases after watching the horror movie.

### 2.4. Analysis of Eye Blinking Rate Variation

**Figure 9:**
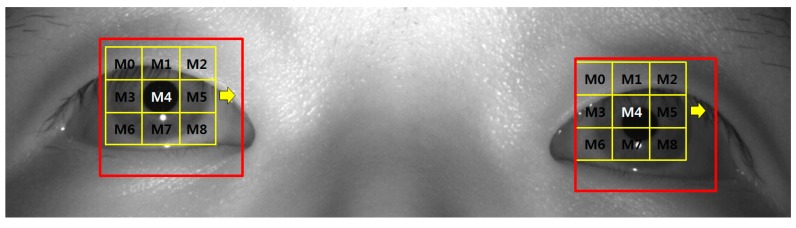
Example of detecting pupil regions using sub-block-based template matching.

**Figure 10:**
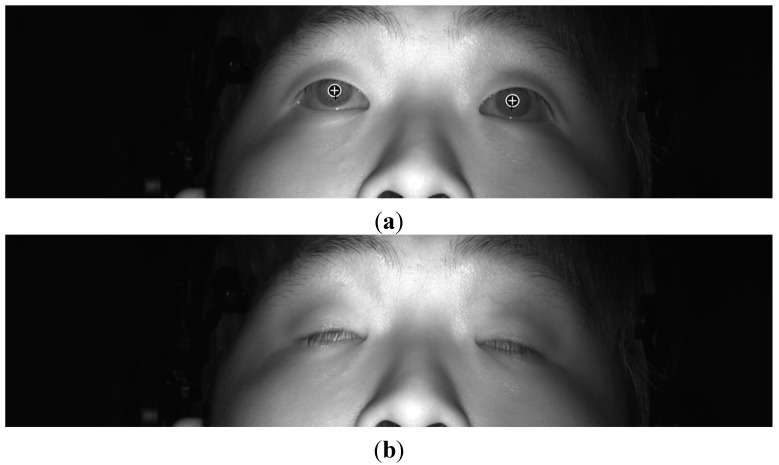
Example of determining whether eyes are open and closed. (**a**) Open eyes; (**b**) closed eyes.

To measure the change of eye blinking rate according to fear, our system used a high-performance (high-speed mega-pixel) camera [20]. We refer to the existing research [27] into detecting pupils and measuring eye blinking rate. Firstly, the corneal specular reflection (SR) is located using image binarization. Based on the detected region of the corneal SR, the ROI for pupil detection is defined as shown in the red boxes of Figure 9. Within these two ROIs, pupil areas are located by sub-block-based template matching. A 3 × 3 mask, including nine sub-blocks (M0–M8) is used for the sub-block-based template-matching algorithm, as shown in Figure 9. Using the sub-block-based template-matching, the pupil region is approximately located. Then, the accurate locations of the boundary and center of the pupil are determined using image binarization, boundary detection and an ellipse-fitting method. If the ellipse-fitting method successfully detects the boundary and center of the pupil, our system determines that the user’s eyes are open. If the detection fails, the user’s eyes are determined to be closed, as shown in Figure 10. Finally, our system measures the number of open to closed eyes for a duration of 1 min, which is the eye blinking rate. If the subject keeps his or her eyes closed, the number of open to closed eyes for a duration of 1 min is maintained, and the consequent eye blinking rate becomes smaller than that of normal eye blinking cases. Because these kinds of data are not the correct ones for measuring the fear of the subject, we removed these data in our experiments.

## 3. Experimental Results

A total of 16 subjects (male: 13, female: three) took part in the experiment. They were physically and mentally healthy, and their average age was 26.56 years old (standard deviation of 1.67). We obtained written, informed consent from each participant. The data from each participant were obtained in a room with an average temperature of approximately 26.6 degrees and an average humidity of approximately 51.9%. To increase the fear for each participant while watching the horror movie, experiments were performed with the lights turned off. The luminance of the room was measured at a maximum of 13 lux. We made the experimental horror movie by excerpting the scary parts from horror movies (Shutter (2004) [32] and Silent Hill (2006) [33]).

The data were acquired using (simultaneously) two desktop computers and a laptop computer. The desktop computer, which was used to obtain the images of the eyes using the high-speed camera [20], had a 3.07-GHz CPU (Intel (R) Core (TM) i7 CPU 950) and 6 GB of RAM. The desktop computer, which was used to obtain the EEG signals by using Emotiv EPOC of the wireless headset type, had a 2.33-GHz CPU (Intel (R) Core (TM) 2 Quad CPU Q8200) and 4 GB of RAM. The laptop computer, which was used to obtain the thermal and visible images by using dual cameras, had a 2.8-GHz CPU (Intel (R) Core (TM) i5-4200H CPU) and 8 GB of RAM. These computers saved the data on a solid-state drive (SSD) for fast acquisition of the data. The proposed method to measure fear was implemented using a C++ program with the Microsoft Foundation Class (MFC) and OpenCV library (Version 2.3.1).

**Figure 11:**
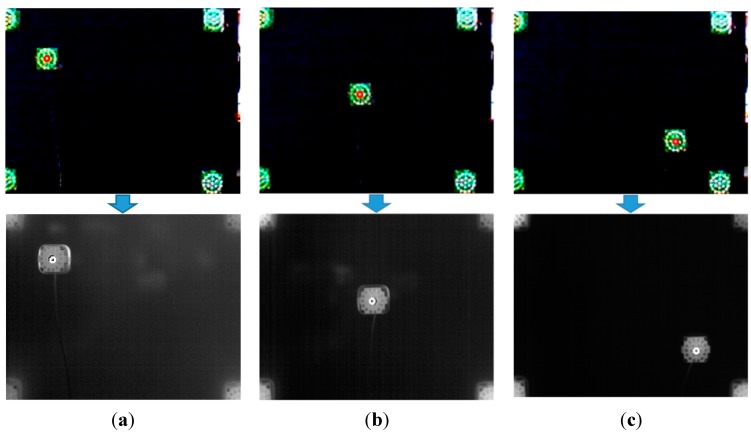
Experiment for measuring the accuracy of the geometric transform. The top and bottom figures of (**a**–**c**) are images from the visible-light and thermal cameras, respectively. The NIR illuminator is positioned at example positions: (**a**) Position 1, (**b**) Position 5 and (**c**) Position 9.

In the first experiment, we measured the accuracy of the calibration of the visible-light and thermal cameras. As explained in Section 2.2 and shown in Figure 5, four NIR illuminators at the four corner positions are used for obtaining the matrix of the geometric transform. Then, we measured the accuracy of the geometric transform using additional NIR illuminators at nine other positions (other than the four corner positions). Figure 11 shows three example cases of NIR illuminator location.

The measured accuracy of the geometric transform between the visible-light and thermal cameras is listed in Table 2. The accuracy is evaluated in terms of the root-mean-square (RMS) error between the ground truth positions and the calculated positions in the thermal image. The ground truth positions are manually obtained from the thermal image. As indicated in Table 2, the average RMS error is approximately 1.18 pixels; we can confirm that the defined facial feature regions (converted from the visible-light image to the thermal image using the geometric transform, as shown in Figure 6 and Figure 7) are accurate.

**Table 2:** Accuracy of geometric transform between visible-light and thermal images.

Ground Truth Position			Calculated Position (by Geometric Transform Matrix)		RMS Error (Pixels)
Position	X	Y	X	Y	
1	62	57	63	58	1.41
2	155	57	156	59	2.24
3	249	61	249	63	2
4	68	112	67	112	1
5	155	112	154	112	1
6	239	111	239	113	2
7	67	176	67	175	1
8	158	178	158	178	0
9	249	179	249	179	0
Average					1.18

**Figure 12:**
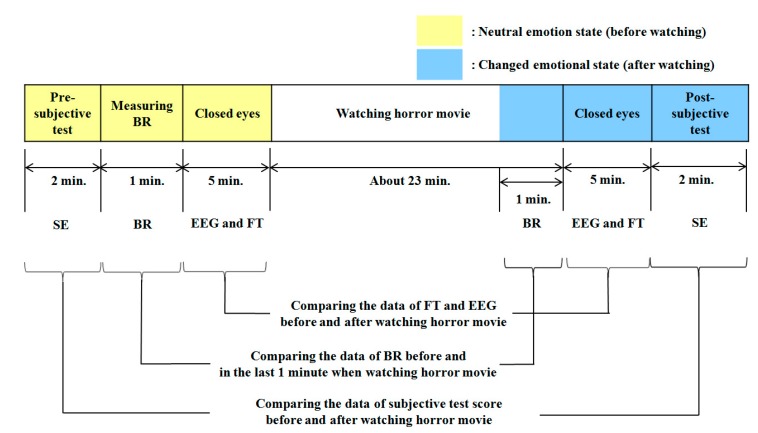
Experimental procedure for measuring fear (BR is blinking rate, FT is facial temperature and SE means subjective evaluation).

Figure 12 shows the experimental procedure for acquiring data to measure fear. To accurately measure the change in fear, the data for EEG signals, facial temperature, eye blinking rate and score on the subjective evaluation were acquired before and after watching the horror movie. The subjective evaluation score was acquired using a questionnaire that included the five questions shown in Table 3. The five questions of Table 3 were developed based on previous studies [34]. Each participant gave the answer to each question on a scale from 1 to 10 points. One and 10 points mean the minimum and maximum levels, respectively.

**Table 3:** Contents of the questionnaire for the subjective test.

Questions for Subjective Test
I am having difficulty seeing
I am scared
I have a headache
I am anxious
I feel unpleasant

**Figure 13:**
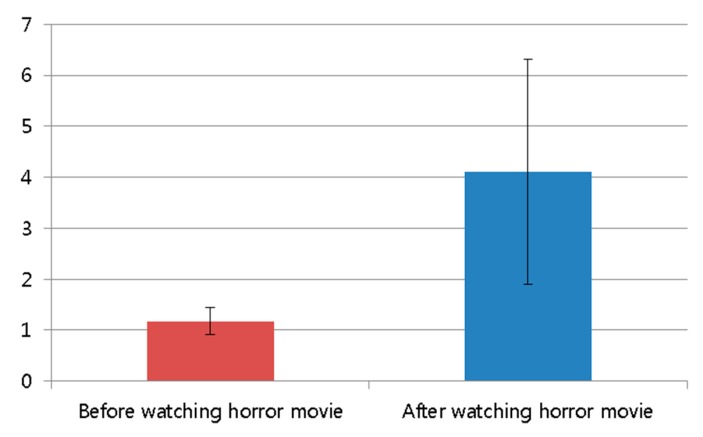
Comparison of subjective evaluation scores before and after watching the horror movie.

The average score of the subjective test after watching the horror movie was higher than that before watching the movie, as shown in Figure 13 and Table 4. The statistical analysis was conducted using an independent unequal variance, two-sample *t*-test [35], which is typically used for hypothesis testing. The calculated *p*-value from the *t*-test is 0.000092, which is less than 0.01 (a confidence level of 99%). From that, the null-hypothesis (that the subjective evaluation scores are the same before and after watching the horror movie) can be rejected. The two subjective evaluation scores before and after watching the horror movie are significantly different at a confidence level of 99%. In addition, we can confirm that the horror movie used in our experiment is effective for generating fear in the participants.

**Table 4:** Average value and standard deviation of subjective evaluation scores.

	Before Watching the Horror Movie	After Watching the Horror Movie
Average	1.175	4.113
Standard deviation	0.272	2.208

**Figure 14:**
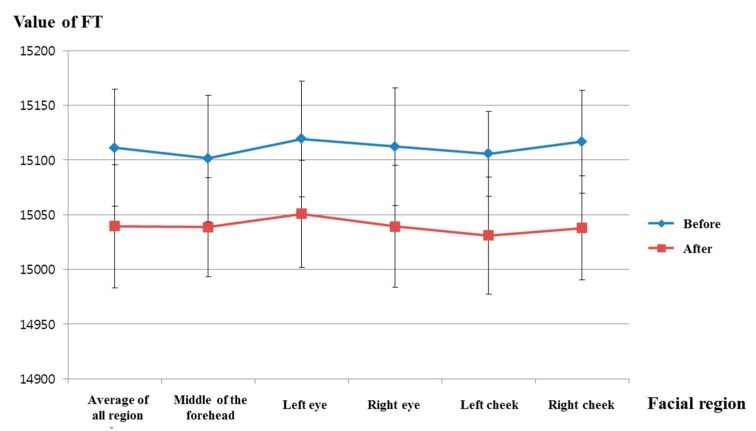
Comparisons of FTs of facial feature regions before and after watching the horror movie (FT is facial temperature).

Figure 14 and Table 5 show the facial temperature of facial feature regions before and after watching the horror movie. Figure 14 indicates a decrease in facial temperature after watching the horror movie in all facial feature regions. The calculated *p*-value for facial temperature before and after watching the movie is 0.00017 in the average of all regions, which is less than 0.01 (a confidence level of 99%). Therefore, we can confirm that the facial temperature is significantly reduced after watching the horror movie, at a confidence level of 99%. In addition, the facial temperature of the right cheek region has the lowest *p*-value (at 0.00006), as shown in Table 5. Our results regarding the decrease in facial temperature in the case of fear are consistent with the results of previous research [12].

**Table 5:** Average, standard deviation and *p*-value of the facial temperature for each facial feature region.

Region	Average of All Regions		Middle of the Forehead		Left Eye		Right Eye	
	Before	After	Before	After	Before	After	Before	After
Average	15,111.08	15,039.47	15,101.48	15,038.56	15,119.34	15,050.73	15,112.23	15,039.24
Standard deviation	46.77801	47.57241	53.48507	56.4484	57.7577	45.40563	53.06459	48.92598
*p*-value	0.00017		0.00295		0.00085		0.00034	
Region	Left cheek		Right cheek					
	Before	After	Before	After				
Average	15,105.64	15,031.03	15,116.71	15,037.77				
Standard deviation	53.81195	55.63021	38.96623	53.48883				
*p*-value	0.00057		0.00006					

**Figure 15:**
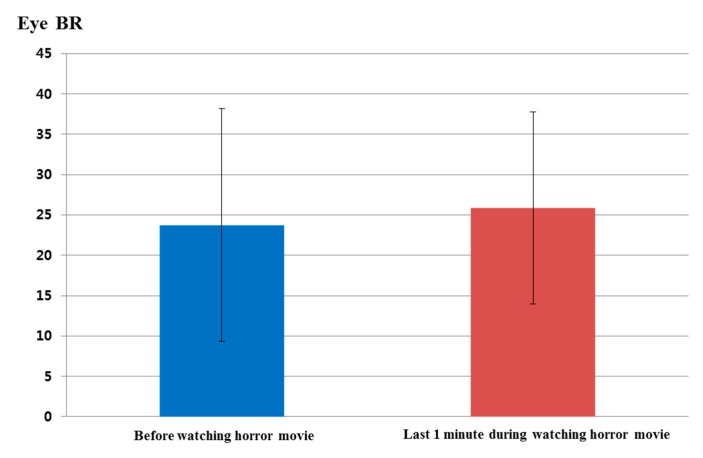
Comparisons of eye blinking rate before watching the horror movie and in the last 1 min of watching the movie (BR is blinking rate).

Figure 15 and Table 6 show the analyzed eye blinking rate before watching the horror movie and during the last 1 min of watching the movie. As shown in Figure 15, the eye blinking rate increased in the last 1 min while watching the horror movie compared to the eye blinking rate before watching it. The calculated *p*-value by the *t*-test is 0.6533, which is larger than the significance levels of 99% (0.01) or 95% (0.05). Therefore, eye blinking rate did not show a statistically-significant difference before watching the horror movie and in the last 1 min while watching.

**Table 6:** Average values and standard deviations of the eye blinking rate.

	Before Watching the Horror Movie	Last 1 Min While Watching the Horror Movie
Average	23.75	25.88
Standard deviation	14.45	11.92

**Figure 16:**
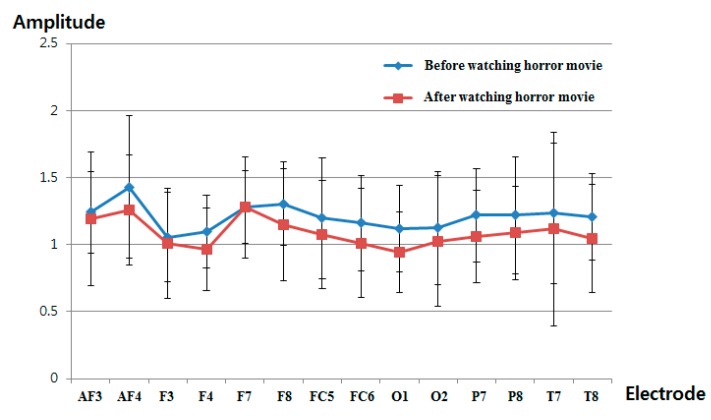
Ratios of delta band to beta band of EEG data before and after watching the horror movie.

Figure 16 and Table 7 show the measured delta band to beta band ratio of each electrode before and after watching the horror movie. As explained in Section 2.3 and shown in Figure 8, we find that the power ratio between delta and beta bands decreases after watching the horror movie. As shown in Figure 16 and Table 7, the delta band to beta band ratio at all electrodes is reduced after watching the horror movie. Whether an electrode shows a significant change before and after watching the movie is based on the *p*-value of the *t*-test. The selected O1 electrode has the lowest *p*-value at 0.1166, as shown in Table 7; however, this is larger than the significance levels of 99% (0.01) or 95% (0.05). Therefore, the EEG signal does not exhibit a significant difference before and after watching the horror movie.

**Table 7:** Average, standard deviation and *p*-value of the EEG signal for all electrodes.

Electrode	AF3		AF4		F3		F4	
	Before	After	Before	After	Before	After	Before	After
Average	1.2417	1.1899	1.4300	1.2577	1.0548	1.0091	1.0962	0.9638
Standard deviation	0.3031	0.4990	0.5296	0.4095	0.3325	0.4124	0.2735	0.3069
*p*-value	0.7256		0.3120		0.7324		0.2074	
Electrode	F7		F8		FC5		FC6	
	Before	After	Before	After	Before	After	Before	After
Average	1.2765	1.2769	1.3056	1.1483	1.1958	1.0737	1.1592	1.0113
Standard deviation	0.3760	0.2711	0.3097	0.4150	0.4526	0.4058	0.3546	0.4092
*p*-value	0.9974		0.2344		0.4281		0.2836	
Electrode	O1		O2		P7		P8	
	Before	After	Before	After	Before	After	Before	After
Average	1.1204	0.9422	1.1249	1.0264	1.2184	1.0587	1.2211	1.0875
Standard deviation	0.3214	0.3023	0.4222	0.4904	0.3464	0.3433	0.4367	0.3492
*p*-value	0.1166		0.5473		0.2003		0.3473	
Electrode	T7		T8					
	Before	After	Before	After				
Average	1.2337	1.1158	1.2095	1.0458				
Standard deviation	0.5250	0.7247	0.3232	0.4044				
*p*-value	0.6026		0.2160					

## 4. Analyses of Experimental Results

As another statistical analysis method, we analyzed the difference between before and after watching the horror movie based on an effect size in the statistics method. In statistics, the effect size has been widely used for indicating the power of an observed phenomenon, and it is generally accepted as a descriptive statistic [36]. Based on previous research [27], we defined Cohen’s *d* values of 0.2, 0.5 and 0.8 as small, medium and large, respectively. Table 8 shows that Cohen’s *d* values represent the difference between two means (before and after watching the horror movie) divided by the standard deviation of the data. The effect size is determined according to a range of value of calculated Cohen’s *d* values: “small” is in the range of 0.2–0.3; “medium” is approximately 0.5; “large” is in the range of 0.8 to infinity [36]. For instance, in Table 8, Cohen’s *d* value of eye blinking rate is 0.1605, which is closer to 0.2 (a small effect) than 0.5 (a medium effect). Therefore, we estimate that the difference in the eye blinking rate (before and in the last 1 min during watching horror movie) is a small effect. As another example, Cohen’s *d* value of the EEG (delta/beta) before and after watching the horror movie was calculated. Because the *p*-value for the O1 node is smaller than those of the other nodes (as shown in Table 7), the EEG value from the O1 node was used for the calculation of Cohen’s *d* value. Cohen’s *d* value of the EEG data is 0.5713, which is closer to 0.5 than either 0.2 or 0.8. Thus, we determine that the difference in EEG data before and after watching the horror movie is a medium effect. In the same manner, the effect sizes of subjective evaluation and facial temperature are large. Based on the *p*-value and Cohen’s *d* value, we can find that the significance of difference of subjective evaluation before and after watching a horror movie is the largest, and that of facial temperature is the second largest one. In addition, EEG and eye blinking rate are the third and fourth ones, respectively. The reason why the significance of EEG is lower than that of subjective evaluation and facial temperature is that the head and facial muscle movements cause noise in the EEG signals.

**Table 8:** Calculated value of Cohen’s *d* before and after watching the horror movie (in the case of eye blinking rate, the calculated value of Cohen’s *d* is based on a comparison before and in the last 1 min while watching the horror movie, as shown in Figure 12).

	Cohen’s *d*	Effect Size
Eye blinking rate	0.1605	Small
EEG	0.5713	Medium
Subjective evaluation	1.8675	Large
Facial temperature	1.6868	Large

As the next analysis, we calculated the R^2^, gradient and correlation values between two data points for eye blinking rate, EEG (delta/beta), facial temperature and subjective evaluation, as shown in Table 9. Because the *p*-value of the O1 node is smaller than those of other nodes (as shown in Table 7), the EEG value from the O1 node was used for the calculation of the R^2^, gradient and correlation values. A best-fit line is calculated by linear regression, and the gradient and R^2^ value were calculated from the fitted line. If the regression line is reliably fitted to the data, the value of R^2^ increases. The range of correlation values is from −1 to 1. If the correlation value is close to zero, the data are uncorrelated [37]. Facial temperature and EEG decreased after watching the horror movie, as shown in Figure 14 and Figure 16, whereas subjective evaluation and blinking rate increased, as shown in Figure 13 and Figure 15. Therefore, facial temperature and EEG data were multiplied by −1 to allow comparison with subjective evaluation and blinking rate. As shown in Table 9, the R^2^ value and correlation value between EEG and facial temperature are the highest, and those between EEG and blinking rate are the lowest. EEG and blinking rate are almost completely uncorrelated, because the correlation value between EEG and blinking rate is close to zero.

**Table 9:** Gradient, R^2^ and correlation values between two modalities.

	Gradient	R^2^	Correlation
EEG *vs.* blinking rate	−0.0063	0.00004	−0.0061
EEG *vs.* facial temperature	0.5965	0.3113	0.5579
EEG *vs.* subjective evaluation	0.1139	0.0085	0.0921
Blinking rate *vs.* facial temperature	0.1329	0.0166	0.1289
Blinking rate *vs.* subjective evaluation	0.5952	0.2491	0.4991
Facial temperature *vs.* subjective evaluation	0.5765	0.2486	0.4986

Table 10 shows the confusion matrix of correlation values among the modalities. In addition, we take the sum of all correlation values except for the auto-correlation value of one (for example, the correlation value between blinking rate and blinking rate) to quantitatively evaluate the individual correlations and the consistency of one modality with others. As shown in Table 10, the sum of all correlation values between facial temperature and other modalities is the highest. That between subjective evaluation and other modalities is the second highest, whereas that between blinking rate and other modalities is the lowest. Based on the results for *p*-values, Cohen’s *d* values and the sum of the correlation values with other modalities, we find that facial temperature and subjective evaluation are more reliable than EEG and blinking rate for the evaluation of fear.

**Table 10:** Confusion matrix and the sum of correlation values between modalities.

	EEG	Blinking Rate	Facial Temperature	Subjective Evaluation	The Sum of All of the Correlation Values with Other Modalities
EEG	1	−0.0061	0.5579	0.0921	0.6439
Blinking rate	−0.0061	1	0.1289	0.4991	0.6219
Facial temperature	0.5579	0.1289	1	0.4986	1.1854
Subjective evaluation	0.0921	0.4991	0.4986	1	1.0898

In our experiment, we already performed the experiments with the video clip having the same length and emotionally-neutral content to the subjects before performing the experiments with the horror movie. It is usually difficult to objectively define the video clip of emotionally-neutral content to the subjects. Therefore, in order to guarantee the credibility of neutral content, 203 images of emotionally-neutral content were selected from the International Affective Picture System (IAPS), because the IAPS images have been widely used as the experimental image for causing a variety of emotions in subjects [10,13,38]. According to the instruction by the IAPS, the experimental images of the IAPS cannot be shown in our paper [38]. With the 203 images from the IAPS, we made the video clip having the same length (about 23 min, as shown in Figure 12) by iterating these images. Through the experiments with the same 16 subjects (which participated in the previous experiments of measuring fear emotion) and the same experimental protocol of Figure 12, we obtained the following four results.

**Figure 17:**
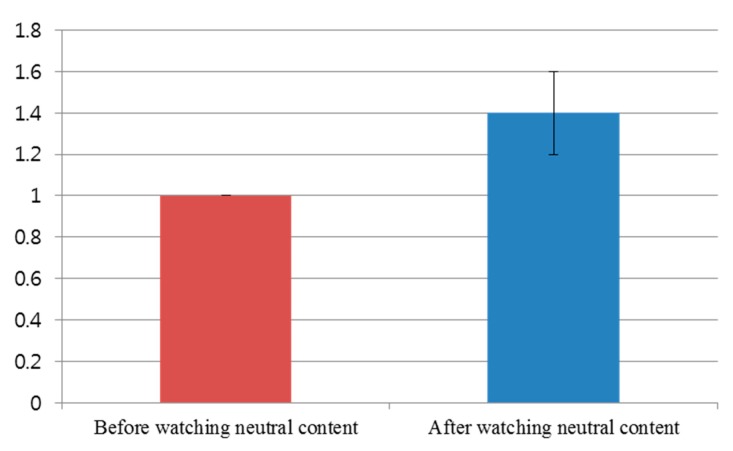
Comparison of subjective evaluation scores before and after watching the video clip of emotionally-neutral content to the subjects.

Figure 17 shows the comparison of subjective evaluation scores before and after watching the video clip of emotionally-neutral content to the subjects. For the subjective evaluation of Figure 17, we used the same questionnaire of Table 3 with the same scale (from 1 to 10) of score. As shown in Figure 17, the average score (about 1.4) of the subjective test after watching the video clip was similar to that (about 1.0) before watching the video clip. However, the average score (about 4.1) of the subjective test after watching the horror movie was much different from that (about 1.2) before watching the horror movie, as shown in Figure 13. From this, we can find that the difference (about 2.9) between the scores of the subjective test before and after watching the horror movie is much larger than that (about 0.4) before and after watching the video clip of neutral emotion.

For proving this conclusion, a statistical analysis was conducted using an independent unequal variance, two-sample *t-*test [35]. The calculated *p*-value by the *t*-test is 0.07418, which is larger than the significance levels of 99% (0.01) or 95% (0.05). From that, the null-hypothesis (that the subjective evaluation scores are the same before and after watching the video clip of neutral content) cannot be rejected. Therefore, we can conclude that the subjective evaluation cannot show a statistically-significant difference before and after watching the video clip of emotionally-neutral content.

**Figure 18:**
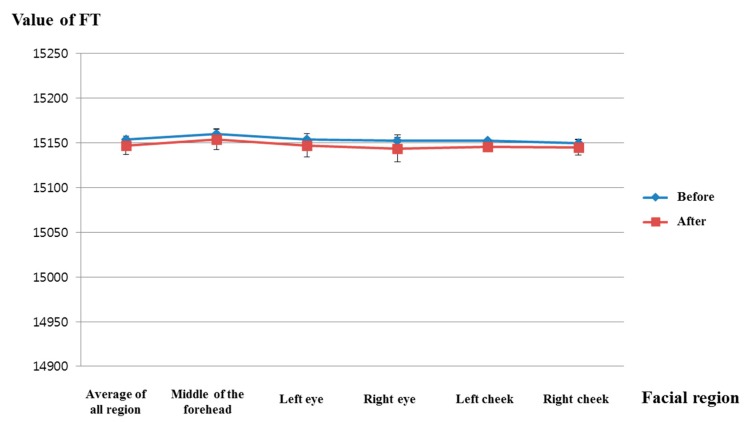
Comparisons of the facial temperature of facial feature regions before and after watching the video clip of emotionally-neutral content to the subjects (FT is facial temperature).

Figure 18 shows the comparison of facial temperatures before and after watching the video clip of emotionally-neutral content to the subjects. Because the change of facial temperature of the right cheek was used in previous experiments of measuring fear emotion due to its lowest *p*-value, as shown in Table 5, the change of facial temperature of the right cheek is also compared in the experiment using the video clip of neutral content. In addition, the same scale of facial temperature values was used for a fair comparison. As shown in Figure 18, the average value (about 15,145) of the facial temperature after watching the video clip was similar to that (about 15,149.6) before watching the video clip. However, the average value (about 15,037.8) of the facial temperature after watching the horror movie was much different from that (about 15,116.7) before watching the horror movie, as shown in Figure 14. From this, we can find that the difference (about 78.9) between the average values of the facial temperature before and after watching the horror movie is much larger than that (about 4.6) before and after watching the video clip of neutral emotion.

For proving this conclusion, the statistical analysis was conducted using an independent unequal variance, two-sample *t-*test. The calculated *p*-value by the *t*-test is 0.476, which is larger than the significance levels of 99% (0.01) or 95% (0.05). From that, the null-hypothesis (that the facial temperatures are the same before and after watching the video clip of neutral content) cannot be rejected. Therefore, we can conclude that the facial temperature cannot show a statistically-significant difference before and after watching the video clip of emotionally-neutral content.

**Figure 19:**
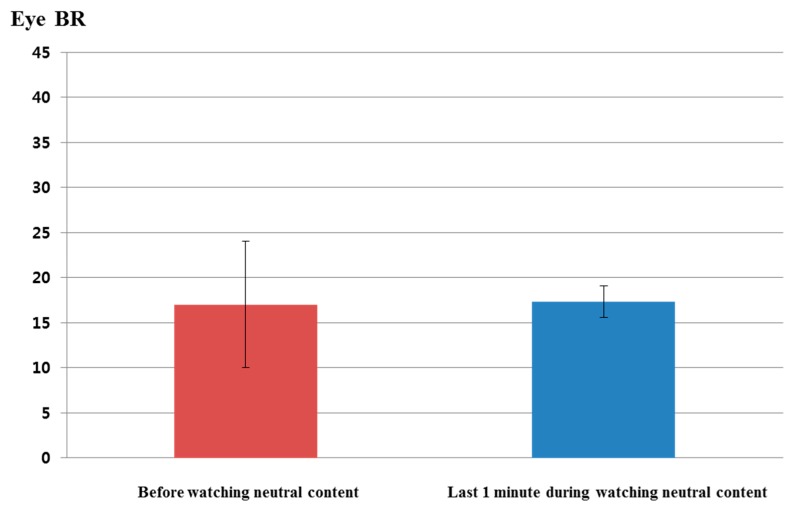
Comparisons of eye blinking rate before and in the last 1 min of watching the video clip of emotionally-neutral content to the subjects (BR is blinking rate).

Figure 19 shows the comparison of eye blinking rate before and in the last 1 min of watching the video clip of emotionally-neutral content to the subjects. For a fair comparison, eye blinking rate is measured as the number of open to closed eyes for a duration of 1 min like the previous experiments for measuring the fear emotion. As shown in Figure 19, the average value (about 17.3) of the eye blinking rate in the last 1 min of watching the video clip was similar to that (about 17) before watching the video clip. However, the average value (about 25.9) of the eye blinking rate in the last 1 min of watching the horror movie was much different from that (about 23.8) before watching the horror movie, as shown in Figure 15. From this, we can find that the difference (about 2.1) between the average values of the eye blinking rate before and in the last 1 min of watching the horror movie is much larger than that (about 0.3) before and in the last 1 min of watching the video clip of neutral emotion.

For proving this conclusion, the statistical analysis was conducted using an independent unequal variance, two-sample *t-*test. The calculated *p*-value by the *t*-test is 0.9447, which is larger than the significance levels of 99% (0.01) or 95% (0.05). From that, the null-hypothesis (that the eye blinking rates are the same before and in the last 1 min of watching the video clip of neutral content) cannot be rejected. Therefore, we can conclude that the eye blinking rate cannot show a statistically-significant difference before and in the last 1 min of watching the video clip of emotionally-neutral content.

**Figure 20:**
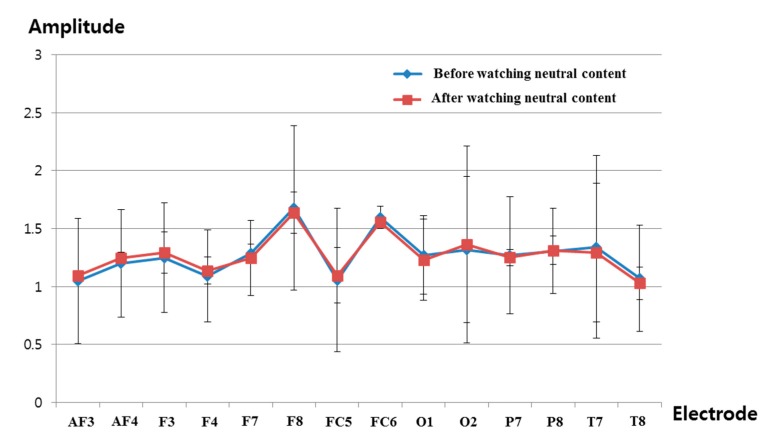
Ratios of delta band to beta band of EEG data before and after watching the video clip of emotionally-neutral content to the subjects.

Figure 20 shows the comparison of the measured delta band to beta band ratio of the EEG signal before and after watching the video clip of emotionally-neutral content to the subjects. In previous research [39,40], the occipital lobe has been reported as the visual processing center of the brain, and the electrodes of O1 and O2 can measure the EEG signals on this occipital lobe based on the international 10–20 system of electrode placement, shown in Figure 4b [26]. In addition, the change of the delta band to beta band ratio of the O1 node was used in previous experiments of measuring the fear emotion due to its lowest *p*-value, as shown in Table 7. Therefore, the change of the delta band to beta band ratio of the O1 node is also compared in the experiment using the video clip of neutral content. The same scale of EEG signal was used for a fair comparison. As shown in Figure 20, the average value (about 1.23) of the change of the delta band to beta band ratio (O1 node) after watching the video clip was similar to that (about 1.27) before watching the video clip. However, the average value (about 0.94) of the change of the delta band to beta band ratio (O1 node) after watching the horror movie was much different from that (about 1.12) before watching the horror movie, as shown in Figure 16. From this, we can find that the difference (about 0.18) between the average values of the change of the delta band to beta band ratio (O1 node) before and after watching the horror movie is much larger than that (about 0.04) before and after watching the video clip of neutral emotion.

For proving this conclusion, the statistical analysis was conducted using an independent unequal variance, two-sample *t-*test. The calculated *p*-value by the *t*-test is 0.917563, which is larger than the significance levels of 99% (0.01) or 95% (0.05). From that, the null-hypothesis cannot be rejected. Therefore, we can conclude that the change of the delta band to beta band ratio cannot show a statistically-significant difference before and after watching the video clip of emotionally-neutral content.

By comparing the results of Figure 13, Figure 14, Figure 15 and Figure 16 with those of Figure 17, Figure 18, Figure 19 and Figure 20, we can conclude that the results of Figure 13, Figure 14, Figure 15 and Figure 16 are caused by the fear emotion. In order to obtain more accurate values in the experiments for measuring the fear emotion excluding other factors, we originally compensated for the changes before and after (in the last 1 min) watching the horror movie by using those before and after (in the last 1 min) watching the video clip of emotionally-neutral content of Figure 17, Figure 18, Figure 19 and Figure 20, from which the results of Figure 13, Figure 14, Figure 15 and Figure 16 were obtained.

In our research, we use the four modalities of eye blinking rate, facial temperature, EEG and subjective evaluation. In order to measure the eye blinking rate, the system for capturing the eye image is usually used, which can be categorized into two kinds, such as wearable and non-wearable devices [41,42]. Because the wearable device requires the user to wear the eye capturing system during the image acquisition, it is regarded as an intrusive method. However, the non-wearable device can capture the user’s eye image without requiring the user to wear any device, and it is regarded as a nonintrusive method [41,42]. Because our device for capturing the eye image is also a non-wearable device, as shown in Figure 2, we define it as a nonintrusive system. Like this criterion, the facial temperature is measured using a non-wearable device, as shown in Figure 2 and Figure 3 in our research, and we define it as a nonintrusive system. In previous research, the device for measuring EEG data can be classified into invasive and noninvasive ones [43,44,45,46]. The invasive method receives the EEG signals from the chips (electrode grid), which are actually inserted inside the head. This has the advantage of obtaining accurate data, but has the disadvantage of requiring a surgical operation to insert the chips inside the head. On the other hand, the noninvasive method uses electrodes that are attached to the head’s skin. This has the advantage of measuring the EEG signal without any surgical operation, but has the disadvantage that the noises caused by head movements can be included in the EEG signal. In general, the former and latter methods are regarded as intrusive and nonintrusive methods, respectively [43,44,45,46]. Based on this criterion, we define our EEG measurement system as a nonintrusive one, because our system uses electrodes that are attached to the head’s skin, as shown in Figure 4. In addition, our EEG device can transfer the data through a wireless interface [25], and the inconvenience caused by using a wired device can be reduced in our EEG device.

Each modality of EEG, eye blinking rate, facial temperature and subjective evaluation has limitations for accurately measuring the fear of the subject. Noises and incorrect signals can be included in the EEG signal in the case of the displacement of the EEG electrodes on the scalp caused by head movement or facial expression changes. Incorrect measurement of the eye blinking rate can occur in the case of inaccurate detection of the eye region or people having inherently narrow eyes. Facial temperature can be also affected by the individual variations of physiological traits. In addition, subjective evaluation can be affected by the individual condition of health or preferences. Therefore, we use multimodal measurements of EEG, eye blinking rate, facial temperature and subjective evaluation in order to overcome these limitations of a single modality and enhance the credibility of measuring the fear of each subject.

## 5. Conclusions

In this research, we proposed a method for the evaluation of fear based on the nonintrusive, multimodal measurement of EEG, blinking rate, facial temperature and subjective evaluation. Experimental results based on *t*-tests and effect sizes indicate that the difference of subjective evaluation before and after watching a horror movie is the largest, whereas those of facial temperature, EEG and blinking rate are the second, third and fourth largest, respectively. In addition, the sum of the correlation values of facial temperature with other modalities is the largest, whereas those of subjective evaluation, EEG and blinking rate are the second, third and fourth largest, respectively. Based on this analysis, we can conclude that facial temperature and subjective evaluation are more reliable than EEG and blinking rate for the evaluation of fear. In future work, we will perform a more accurate evaluation of various mental states, such as sadness, happiness and surprise. In addition, we will study methods of combining other modalities, such as ECG and SKT.
